# Transcriptome shifts triggered by vitamin A and *SCD* genotype interaction in Duroc pigs

**DOI:** 10.1186/s12864-021-08244-3

**Published:** 2022-01-07

**Authors:** Emma Solé, Rayner González-Prendes, Yelyzaveta Oliinychenko, Marc Tor, Roger Ros-Freixedes, Joan Estany, Ramona N. Pena

**Affiliations:** 1 Departament de Ciència Animal, Universitat de Lleida – AGROTECNIO-CERCA Center, Av. Rovira Roure 191, 25197 Lleida, Spain; 2 Animal Breeding and Genomics, Wageningen University & Research, 6708PB, Wageningen, The Netherlands; 3 The Institute of Animal Science NAAS, Kharkiv, Ukraine

**Keywords:** *SCD* gene, Fatty acid, Vitamin A, Meat quality, RNA-seq, Swine

## Abstract

**Background:**

The composition of intramuscular fat depends on genetic and environmental factors, including the diet. In pigs, we identified a haplotype of three SNP mutations in the stearoyl-coA desaturase (*SCD*) gene promoter associated with higher content of monounsaturated fatty acids in intramuscular fat. The second of these three SNPs (rs80912566, C > T) affected a putative retinol response element in the *SCD* promoter. The effect of dietary vitamin A restriction over intramuscular fat content is controversial as it depends on the pig genetic line and the duration of the restriction. This study aims to investigate changes in the muscle transcriptome in *SCD* rs80912566 TT and CC pigs fed with and without a vitamin A supplement during the fattening period.

**Results:**

Vitamin A did not affect carcass traits or intramuscular fat content and fatty acid composition, but we observed an interaction between vitamin A and *SCD* genotype on the desaturation of fatty acids in muscle. As reported before, the *SCD*-TT pigs had more monounsaturated fat than the *SCD*-CC animals. The diet lacking the vitamin A supplement enlarged fatty acid compositional differences between *SCD* genotypes, partly because vitamin A had a bigger effect on fatty acid desaturation in *SCD*-CC pigs (positive) than in *SCD*-TT and *SCD*-TC animals (negative). The interaction between diet and genotype was also evident at the transcriptome level; the highest number of differentially expressed genes were detected between *SCD-*TT pigs fed with the two diets. The genes modulated by the diet with the vitamin A supplement belonged to metabolic and signalling pathways related to immunity and inflammation, transport through membrane-bounded vesicles, fat metabolism and transport, reflecting the impact of retinol on a wide range of metabolic processes.

**Conclusions:**

Restricting dietary vitamin A during the fattening period did not improve intramuscular fat content despite relevant changes in muscle gene expression, both in coding and non-coding genes. Vitamin A activated general pathways of retinol response in a *SCD* genotype-dependant manner, which affected the monounsaturated fatty acid content, particularly in *SCD*-CC pigs.

**Supplementary Information:**

The online version contains supplementary material available at 10.1186/s12864-021-08244-3.

## Introduction

Intramuscular fat (IMF) content and its composition affect overall pork acceptability by influencing organoleptic attributes such as flavour, texture, and juiciness. Meat quality can be improved by direct husbandry practices, for instance through adapting the diet to meet production aims [1]. In this context, vitamin A restriction during the fattening period can improve IMF content and marbling score in beef cattle (reviewed in [2]). This effect is induced by retinoic acid, one of the bioactive compounds of the vitamin A family, which prompts two waves of effects over preadipocyte differentiation [3, 4]. On the one hand, it stimulates preadipocyte differentiation during the early commitment of embryonic stem cells into the adipocyte lineage but, at later stages, it has an inhibitory role over the terminal differentiation of preadipocytes. Thus, restriction of vitamin A or their precursors (β-carotenes) at early fattening stages (14-22 months of age in beef cattle) had a prominent effect over IMF content through enhanced hyperplasia of adipocytes [2].

In pigs, however, the effect of vitamin A restriction is controversial. The IMF of pigs fed a control feed (7500 IU vitamin A /kg) did not differ from pigs supplemented with > 10 times more vitamin A (100,000 IU/kg) [5]. Other authors have reported increases in IMF content with diets restricted in vitamin A [6–10], although the magnitude (and even the direction) of this effect depends on the genetic type of the pigs [11]. In contrast, the effect of vitamin A on IMF fatty acid (FA) composition is more robust across experiments. Vitamin A restriction promotes deposition of monounsaturated FAs (MUFA) and raises the desaturation index of fat [5, 7].

Intramuscular fat content and its composition are traits of moderate-to-high (range 0.26 to 0.86) heritability [12] and can therefore be improved through selection programs. As a result from a previous genome-wide association study (GWAS) using IMF content and composition data from muscle (*gluteus medius* and *longissimus thoracis*) and subcutaneous fat from a line of commercial Duroc pigs, we identified two genomic regions with prominent but distinct effects on IMF content (in SSC6) and on MUFA, notably oleic acid (in SSC14) [13]. These two regions co-located with the position of the leptin receptor (*LEPR*) and stearoyl-coA desaturase (*SCD*) genes, respectively. The *SCD* gene encodes a limiting enzyme in the biosynthesis of MUFA. In a previous work, we identified three linked SNP mutations in the promoter of this gene [14]. The haplotype H1, corresponding to the C-T-A combination of alleles, associated with a higher content of MUFA in IMF and subcutaneous fat regarding the alternative H2 haplotype (T-C-G). The middle SNP (rs80912566, C > T) changes a potential binding site for retinoid X receptors (RXR) [14]. Two families of the retinoic acid receptors (RAR and RXR) mediate signals in multiple physiological processes, including the modulation of genes involved in adipogenesis, mitochondria and lipid metabolism, through their interaction with retinoic acid compounds [15]. However, on the other hand, no relationship has been described between the *LEPR* activity and vitamin A.

Given all the above, we have investigated here the effect of the interaction between dietary vitamin A content and the *SCD* genotype on IMF content and FA composition. A global transcriptome sequencing approach was used to characterise the changes in gene expression triggered by this interaction in pigs fed different levels of vitamin A supplement.

## Material and methods

### Animals and experimental design

All pigs used in the study were raised and slaughtered in commercial units following applicable regulations and good practice guidelines on the protection of animals kept for farming purposes during transport and slaughter. All experimental procedures were approved by the Ethics Committee for Animal Experimentation of the University of Lleida (agreement CEEA 05-04/15). This study is reported in accordance with the ARRIVE guidelines.

In a first experiment to study the effect of the interaction between dietary vitamin A content and the *SCD* genotype on carcass and IMF content and FA composition, 108 Duroc barrows were reared in two batches and were maintained under the same rearing conditions with ad libitum access to feed (Additional file 1: Table S1). This population was generated by mating 59 sows with 28 boars and randomly choosing, on average, two barrows per litter. At 30 kg of live-weight, pigs were randomly assigned to two dietary treatments (Table 1 and Additional file 1: Table S2): a standard feed supplemented with vitamin A (VA+), or the same diet without supplementation (VA-).

**Table 1:** Retinyl acetate content in the feed used in this study

Diet	Phase	Age	Retinyl acetate (mg/kg feed)
**With vitamin A supplement (VA+)**	STARTER	80-110 d	3.125
	GROWTH	110-160 d	3.580
	FINISHER	160-210 d	2.440
**Without vitamin A supplement (VA-)**	STARTER	80-110 d	0.275
	GROWTH	110-160 d	0.725
	FINISHER	160-210 d	0.395

Animals were slaughtered at 207 ± 7 days and 130 ± 11 kg of body weight in a commercial slaughterhouse equipped with a carbon dioxide stunning system. Measured body composition traits included carcass yield, loin thickness and backfat thickness between the third and fourth last ribs by using an ultrasound automatic scanner (AutoFOM, SFK-Technology, Denmark). The carcass lean percentage was estimated based on 35 measurements of AutoFOM points by using the official approved equation (Decision 2001/775/CE, 2001). Samples of *semimembranosus* muscle were collected immediately after slaughter and snap frozen in dry ice. After chilling for about 24 h at 4 °C, samples of the *gluteus medius* and *longissimus thoracis* were collected and stored at − 80 °C until required.

In a second experiment to study changes in muscle transcriptome in the pigs from the first experiment, 40 pigs from the same batch were selected using a factorial design balanced for diet (VA+ and VA-) and *SCD* genotypes (TT and CC) (Additional file 1: Table S2).

### Fatty acid analysis

Fatty acid content was analysed in a representative sample from pulverized freeze-dried muscle. For each sample, the IMF content and FA composition were determined in duplicate by gas chromatography [16]. Fatty acid methyl esters were obtained by transesterification using a solution of 20% boron trifluoride in methanol [17]. Methyl esters were determined by gas chromatography using a SP2330 capillary column (30 m by 0.25 mm; Supelco Inc., Bellefonte, PA). The quantification was performed through area normalization after adding into each sample 1,2,3-tripentadecanoylglycerol as an internal standard. The proportion of individual FA, saturated FA (SFA), polyunsaturated FA (PUFA) and MUFA were calculated as percentages relative to total FA content. Intramuscular fat content was predicted as the sum of each individual FA expressed as triglyceride equivalents [18].

### Statistical analyses for carcass and fatty acid data

Data on carcass traits and FA composition were analysed using a linear model that included the rearing batch (2 levels), diet (VA+ and VA-), the *SCD* genotype (TT, TC, CC), and the interaction of diet by *SCD* genotype. The *LEPR* genotype was not included in the model as the number of pigs per genotypes was not balanced (there were very few *LEPR*-TT pigs). The age at slaughter was included as a covariate to analyse carcass traits and IMF content. To analyse FA composition, IMF content was used as a covariate. Multiple pairwise comparisons were performed with a Tukey test setting *P* < 0.05 as significance threshold. Analyses were performed using the statistical package JMP Pro 15 (SAS Institute Inc., Cary, NC).

### DNA isolation and genotyping

Genomic DNA was isolated from muscle samples by incubation with a lysis buffer with proteinase K followed by phenol:chloroform purification using standard protocols [19]. The quantification and purity of DNA was determined in a Nanodrop ND-1000 Spectrophotometer (Thermo Fisher Scientific, Waltham, MA, USA). DNA integrity was tested by electrophoresis in agarose gels.

All samples were genotyped for *SCD* rs80912566 by real time qPCR (QuantStudio3, Applied Biosystems, Waltham, MA, USA) with High Resolution Melt analysis (Luminaris Color HRM Master Mix, Thermo Scientific, Waltham, MA, USA) as in [13].

### RNA isolation and library construction

Total RNA was isolated from *m. semimembranosus* samples using TRI Reagent (Invitrogen, Thermo Scientific, Waltham, MA, USA) and Direct-zol™ RNA Miniprep Plus Kit (Zymo Research, BioSystems, CA, USA) according to the manufacturer’s protocol. This muscle was selected because it was collected at slaughter and yielded RNA with the integrity needed for RNA-seq analysis. RNA integrity number (RIN) and purity were checked by a Bioanalyzer 2100 (Agilent Technologies, CA, USA). RIN was in the range 8.0-9.0.

The RNA samples were sequenced by Centre Nacional d’Anàlisi Genòmica (CNAG-CRG, Barcelona, Spain, http://www.cnag.crg.eu/). Libraries were prepared using the TruSeq SBS v-3HS kit (Ilumina, San Diego, CA) according to the manufacturer’s protocol. Each library was paired-end sequenced (2 × 100 bp) to 65 M reads with phred quality score 80-90% in a Hi-Seq 2000 platform.

### Analysis of RNA-seq transcriptomic data

Quality of the raw sequencing data was assessed with the fastp tool [20]. With this approach, in addition to the default parameters to filtering, reads with quality scores per base lower than 30, shorter than 36 bp or unpaired were removed. Filtered sequences were aligned to the *Sus scrofa* reference genome (Sscrofa11.1) with the STAR 2.5.4b tool [21]. Mapping statuses were analysed with qualimap and plotted with MultiQC v1.0 [22]. After the alignment, reads were counted with the Feature Counts v1.24.1 software [23] and differential expression (DE) analysis between *SCD* genotypes, diets, and their combined groups was performed with the approach implemented in DeSeq2 v.1.14.1 software [24]. Adjusted *p*-values (q-value < 0.10) and fold change (FC) > 1.2 (upregulated) or < 1/1.2 = 0.83 (downregulated) were set as the threshold for significantly different expression. Functional analysis of differentially expressed genes (DEG) was then subjected to gene ontology (GO) functional enrichment analysis, KEGG pathways and targets of transcription factor binding with Enrichr v1.0 software [25]. Visualisation of gene interactions was performed with Cytoscape 3.9.0 and the StringApp connector [26].

In total, an average of 62.4 M (range 48.8-91.5 M) raw reads per sample were generated. After filtering, an average of 58 M of reads were retained for further analysis and 84.8% (range 77.4-86.8%) of the reads were uniquely mapped to the pig reference genome (Sscrofa11.1; Additional file 1: Table S3). Sequencing files are available from NCBI-GEO with access number GSE183909.

### Validation of RNA-seq results by quantitative real-time PCR analysis

Quantitative real time PCR (qPCR) was used to validate ten relevant genes in the list of DEGs. Briefly, 2 μg of total RNA from the 40 pigs in the RNA-seq experiment were retrotranscribed using SuperScript IV retrotranscriptase (Invitrogen, Carlsbad, CA) with oligo-dT and random hexamers. Primers (Additional file 1, Table S4) were designed with the Primer Blast tool using the mRNA reference sequences provided in NCBI GENE, so that they will hybridise to all the transcripts described for each gene. Three reference genes (*HPRT*, *B2M* and *RPL32*), were included in this analysis. For each gene, a standard curve was generated by amplifying serial dilutions of a control cDNA to check for linearity between initial template concentration and Ct values. Quantitative real-time PCR assays were carried out in triplicate in a QuantStudio3 device (Applied Biosystems, Waltham, MA, USA) in a final volume of 8 μl containing 1× Maxima SYBR Green/ROX qPCR Master Mix (Thermo Scientific, Waltham, MA, USA), 200 nM of each primer and 3 μl cDNA template diluted 1:30 in water. The following thermal profile was used for all reactions: 10 min at 95 °C, 40 cycles of 15 s at 95 °C and 30 s at 60 °C, followed by a slow ramp from 60 to 95 °C to generate a dissociation curve to control the specificity of the amplified product. In order to quantify and normalise the expression data we used the ΔΔCt method [27] against the geometric mean of the three reference genes. For each gene, expression values between groups were compared with a t-test and differences were considered significant at *P* < 0.05.

## Results

### Effect of vitamin A supplementation on carcass traits

Carcasses of pigs fed the VA- diet were on average 3.8 kg heavier than those on the VA+ diet (*P* < 0.05; Table 2). However, other carcass traits, including IMF content in three muscles, were not affected by the dietary vitamin A content (*P* > 0.05). On the other hand, pigs with the *SCD* TT genotype had more subcutaneous fat and less loin thickness than CC pigs, which resulted in a lower carcass lean content (*P* < 0.05). There was no significant interaction between the *SCD* genotype and the diet for the tested carcass traits (Table 2).

**Table 2:** Least square means (±SE) for carcass traits and intramuscular fat content by diet and *SCD* rs80912566 genotype. Number of pigs per factor is indicated in parenthesis

	Diet^2^		***SCD*** rs80912566 genotype			***P***-value		
Trait^1^	VA- (54)	VA+ (55)	TT (38)	TC (44)	CC (27)	Diet	***SCD***	Diet×***SCD***
Carcass weight, kg	98.97 ± 1.40^b^	95.17 ± 1.33^a^	97.84 ± 1.51	96.68 ± 1.35	96.69 ± 1.81	0.03	n.s.	n.s.
BFT, mm	34.75 ± 1.07	34.81 ± 1.02	37.18 ± 1.15^a^	34.31 ± 1.03^ab^	32.85 ± 1.38^b^	n.s.	0.03	n.s.
Loin depth, mm	41.28 ± 1.27	40.13 ± 1.21	37.96 ± 1.37^a^	40.98 ± 1.22^ab^	43.17 ± 1.64^b^	n.s.	0.03	n.s.
Lean content, %	35.83 ± 1.19	35.45 ± 1.13	32.63 ± 1.28^b^	36.18 ± 1.14a^b^	38.09 ± 1.54^a^	n.s.	0.01	n.s.
IMF (GM), %	6.27 ± 0.27	6.80 ± 0.26	6.63 ± 0.29	6.42 ± 0.26	6.26 ± 0.35	n.s.	n.s.	n.s.
IMF (LT), %	4.44 ± 0.21	4.41 ± 0.19	4.63 ± 0.22	4.40 ± 0.19	4.24 ± 0.26	n.s.	n.s.	n.s.
IMF (SM), %	3.24 ± 0.41	3.22 ± 0.43	2.78 ± 0.48	3.36 ± 0.39	3.55 ± 0.42	n.s.	n.s.	n.s.

^1^*BFT* Backfat thickness, *IMF* intramuscular fat content, expressed on a wet weight basis, *GM* m. *gluteus medius*, *LT* m. *longissimus thoracis*, *SM* m. *semimembranosus*. Within each row and factor, means with different superscripts differ significantly (*P* < 0.05). n.s. – not significant (*P* > 0.05)

^2^Diets with (VA+) and without (VA-) vitamin A supplement in the feed formulation

### Effect of vitamin A supplementation on IMF composition

**Fig. 1:**
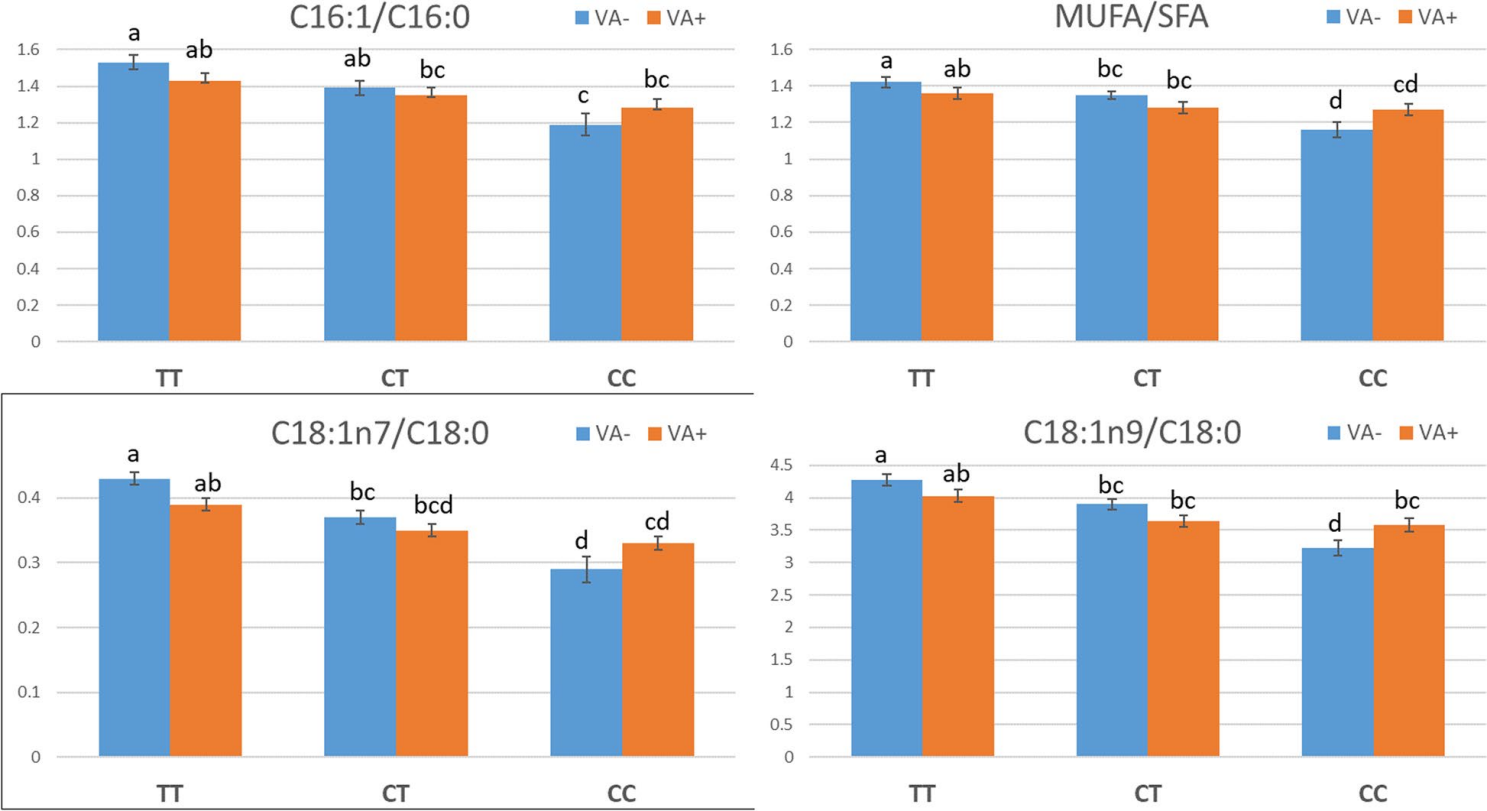
Effect of *SCD* genotype and diet on fatty acid desaturation indexes. Diets with (VA+) and without (VA-) vitamin A supplement in the feed formulation. Within each panel, bars not connected with the same letter differ at *P* < 0.05. Errors bars are SE

The FA composition in the IMF of the three muscles analysed was not affected by the vitamin A supplement. As described before [14], the rs80912566 TT genotype was associated with an increase in the C16:1, C18:1n9 and MUFA content at the expense of the C16:0, C18:0 and SFA, respectively (Table 3 and Additional file 1; Table S5), which led to increased desaturation indexes (C16:1/C16:0, C18:1n9/C18:0 and MUFA/SFA; Fig. 1 and Additional file 2: Fig. S1). There was a clear interaction between the *SCD* genotype and the dietary vitamin A content. This interaction was significant for most FAs related to the *SCD* desaturation pathway in the *m. gluteus medius* (Table 3 and Fig. 1). The VA- diet tended to enlarge the compositional differences between the TT and CC genotypes. Conversely, in the pigs fed the VA+ diet, the differences between genotypes were smaller and sometimes not significant. Vitamin A restriction triggered differential FA compositional changes in TT and CC pigs (*P* < 0.05; Fig. 1). The interaction between vitamin A and the *SCD* genotype was also detected in leaner muscles such as *m. longissimus thoracis* (C18:0, MUFA, C18:1n7/C18:0, C18:1n9/C18:0 and MUFA/SFA, at *P* < 0.05) and *m. semimembranosus* (C18:0 at *P* < 0.05 and SFA at *P* < 0.10) (Additional file 1: Table S5 and Additional file 2: Fig. S1). When the dietary vitamin A was restricted, CC pigs had more saturated FAs, while in TT pigs the FAs that result from SCD desaturation were only slightly affected by the diet. This trend was consistent in the three muscles analysed (Fig. 1 and Additional file 2: Fig. S1) and indicates that, overall, CC pigs were more sensitive to dietary vitamin A than TT pigs in increasing FA desaturation associated with SCD activity.

**Table 3:** Least square means (±SE) for fatty acid composition in the intramuscular fat of *m. gluteus medius* by diet and *SCD* rs80912566 genotype. Number of pigs per factor is indicated in parenthesis

	VA- diet^2^			VA+ diet			***P***-value		
	***SCD*** genotype			***SCD*** genotype			Diet	***SCD***	Diet×***SCD***
FAs^1^	TT (18)	TC (25)	CC (11)	TT (20)	TC (19)	CC (16)			
C16:0, %	24.88 ± 0.28^b^	25.22 ± 0.24^b^	26.57 ± 0.37^a^	25.27 ± 0.28^ab^	25.73 ± 0.28^ab^	25.26 ± 0.32^ab^	n.s.	0.03	< 0.01
C16:1, %	3.80 ± 0.09^a^	3.51 ± 0.08^abc^	3.18 ± 0.12^bc^	3.61 ± 0.09^ab^	3.46 ± 0.09^abc^	3.21 ± 0.10^c^	n.s.	< 0.0001	n.s.
C18:0, %	10.21 ± 0.19^d^	11.01 ± 0.16^bc^	12.51 ± 0.25^a^	10.74 ± 0.19^cd^	11.58 ± 0.19^b^	11.72 ± 0.21^a^	n.s.	< 0.0001	< 0.01
C18:1n7, %	4.33 ± 0.06^a^	4.09 ± 0.05^b^	3.66 ± 0.08^d^	4.16 ± 0.06^ab^	3.98 ± 0.06^bc^	3.79 ± 0.07^cd^	n.s.	< 0.0001	0.05
C18:1n9, %	43.36 ± 0.35^a^	42.84 ± 0.30^ab^	40.55 ± 0.47^c^	42.95 ± 0.35^ab^	41.89 ± 0.36^bc^	41.63 ± 0.40^bc^	n.s.	< 0.0001	0.02
SFA, %	37.09 ± 0.44^c^	38.14 ± 0.38^bc^	41.21 ± 0.59^a^	38.10 ± 0.44^bc^	39.43 ± 0.45^ab^	39.18 ± 0.50^ab^	n.s.	< 0.0001	< 0.01
MUFA, %	52.29 ± 0.42^a^	51.25 ± 0.36^ab^	48.15 ± 0.56^c^	51.53 ± 0.42^ab^	50.11 ± 0.42^bc^	49.40 ± 0.47^c^	n.s.	< 0.0001	0.02
PUFA, %	10.62 ± 0.25^ab^	10.61 ± 0.21^ab^	10.65 ± 0.33^ab^	10.37 ± 0.25^ab^	10.47 ± 0.25^bc^	11.42 ± 0.28^a^	n.s.	0.03	n.s.

^1^FA traits presented as percentage of total FAs in the sample. *SFA* saturated FAs, *MUFA* monounsaturated FAs, *PUFA* polyunsaturated FAs. Within each row, means with different superscripts differ significantly (*P* < 0.05). n.s. – not significant (*P* > 0.05)

^2^Diets with (VA+) and without (VA-) vitamin A supplement in the feed formulation

### Differentially expressed genes between diet and *SCD* groups

**Fig. 2:**
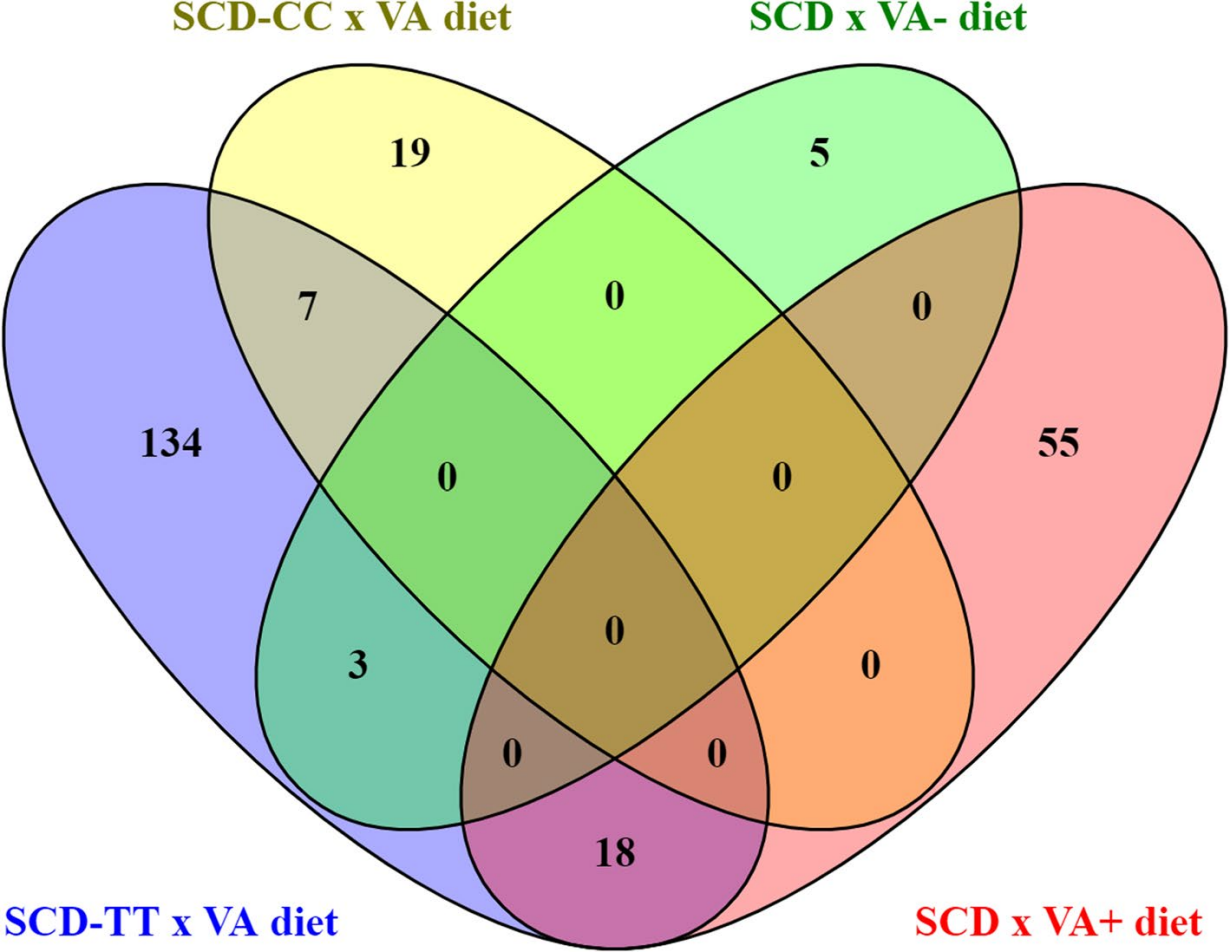
Venn diagram showing the number of overlapping differentially expressed genes between diets (VA+ and VA-) by rs80912566 *SCD* genotype and between *SCD* genotypes (*SCD*-TT and *SCD*-CC) by diet. Dietary treatment was the same but with (VA+) and without (VA-) vitamin A supplement in the feed formulation

The number of DEGs between pigs under different diet treatments or with different *SCD* genotypes was low (43 and 24, respectively), with no overlapping. However, the number of DEGs increased when dietary vitamin A by *SCD* genotype groups were studied (269 transcripts corresponding to 241 unique genes; Table 4). Only 28 of the 241 genes overlapped across the lists of DEGs in the four diet-by-genotype group comparisons (Fig. 2). Most DEGs were classified as messenger RNA from protein coding genes (79%) (Table 4). The second most abundant RNA type was the long non-coding RNA (lncRNA) class, which accounted for 16% of total DEGs detected in this experiment. The full list of DEGs is shown in Additional file 1: Table S6, which includes 202 unique HGNC gene name identifiers (HUGO Gene Nomenclature Committee database).

**Table 4:** Number and classification of differentially expressed genes (n. DEG) by diet, *SCD* genotype and dietary vitamin A by *SCD* genotype groups

Factor	Comparison	n. DEG	coding	lncRNA^**a**^	pseudogene	snoRNA	miRNA	MT tRNA
Diet	VA+ vs VA-	43	27	10		3	3	1
*SCD*	TT vs CC	24	17	7		1		
Diet in *SCD*_TT genotype	TT_VA+ vs TT_VA-	162	134	22	3	1	1	1
Diet in *SCD*_CC genotype	CC_VA+ vs CC_VA-	26	21	1	1	1	1	1
*SCD* genotype in VA+ diet	TT_VA+ vs CC_VA+	73	59	13		1		
*SCD* genotype in VA- diet	TT_VA- vs CC_VA-	8	7			1		

^a^*lncRNA* long non-coding RNA, *pseudogene* transcribed pseudogene, *snoRNA* small nucleolar RNA, *miRNA* microRNA, *MT tRNA* mitochondrial transference RNA

The *SCD* gene was detected as a DEG when the two genotypes (TT vs CC) were compared (FC = 2.15) with boost effect in the diet with vitamin A supplementation (FC = 2.84) but not under the VA- diet (Additional file 1: Table S6). A group of seven genes (*COX15*, *ABCC2*, *CWF19L1*, *ENSSSCG00000048329*, *ENSSSCG00000049992*, *SEC31B* and *FBXW4*) located -500 kb/+ 200 kb from the *SCD* gene were also differentially expressed, which might represent a case of piggyback co-expression due to the close distance between genes [28].

### Functional analysis of DEG

The functional analysis of the full list of DEGs was based on GO terms, KEGG pathways and common transcription factors associated to each gene. Functional classification (Additional file 1: Table S7) revealed an overrepresentation of genes encoding proteins located in membrane-bounded vesicles (25 genes; *P* = 0.007), lipid transport (10 genes; *P* = 0.003) and connective tissue development (9 genes; *P* = 0.002), amongst others. This latter group includes several genes with large fold-change values of differential expression, such as *TNMD* (tenomodulin) and *COMP* (cartilage oligomeric matrix protein), which were 131- and 52-times more expressed in TT fed the VA- diet respect to those fed the VA+ diet. *TNMD* encodes for a potent inhibitor of angiogenesis and *COMP* is a non-collagen extracellular matrix protein that triggers integrin signalling to the cell surface. Their expression has not been related to retinol pathways, so far. Visualisation of protein-to-protein and protein-to-DNA interactions based on the STRING database indicated three main networks of relationships (Additional file 2: Fig. S2). The largest network included 20 genes membrane-bound vesicles (Additional file 2: Fig. S3.A). Additionally, genes involved in the beta-oxidation of FA in peroxisomes also grouped together (*MVK*, *ACCA1*, *ECHDC2*, and peroxins *PEX6* and *PEX5L*) (Additional file 2: Fig. S3.B).

The target transcription factor analysis identified ten transcription factors that can potentially regulate 104 of the 202 unique DEG gene name identifiers (Table 5). These 10 transcription factors, which include proteins activated by retinol (PPARG, PPARA, RARA, RXRA) or by sterols (SREBF1 and SREBF2), are known to interact among themselves in a competitive manner (reviewed in [29]; also [30–32]), which agrees with the partial overlap of regulated genes by transcription factor in Table 5. Functional classification within these 104 genes (Additional file 1: Table S8) revealed an overrepresentation of genes encoding proteins located in membrane-bounded vesicles (*n* = 15; *P* = 0.003), which had a prominent role in endocytosis (*P* = 0.002), in the group of 40 genes regulated by SREBF1 and SREBF2 transcription factors. This classification partially overlapped with the genes regulated by the IRF2, HNF1A and HNF4A transcription factors. The genes regulated by the HNF1A and HNF4A transcription factors also included genes with activity related to lipid metabolic process (7 genes; *P* = 0.02). On the other hand, genes regulated by RARA and RXRA nuclear receptors were involved in a variety of functions, which included immunity and inflammation responses (*P* = 0.02), lipid binding (*P* = 0.03) and apoptosis (*P* = 0.03). Finally, the genes clustered under the regulation of PPARG and PPARA include genes with metal-binding (11 genes, *P* = 0.03) and long-chain FA-modifying (2 genes, *P* = 0.05) capacities.

**Table 5:** Regulators predicted for the set of annotated coding differentially expressed genes

Transcription factor	n	Genes
HMGA1	13	*ACTC1*; *CGGBP1*; *CNST*; *FBXO33*; *IREB2*; *MPI*; *MTTP*; *NEGR1*; *PCDHGA6*; *PDE4D*; *PPM1D*; *PTX3*; *TBXAS1*
HNF1A	12	*ADPRH*; *AGK*; *COL17A1*; *ECE1*; *GPX2*; *ITGAL*; *NAV2*; *PYCARD*; *RNF122*; *STARD8*; *TBXAS1*; *TRAK2*
HNF4A	25	*ABCA1*; *AURKB*; *C12ORF49*; *CDK23*; *CGGBP1*; *CORO1A*; *CRYBB3*; *CYB561D1*; *ECE1*; *ECHDC2*; *GPX2*; *IMPAD1*; *IRX3*; *MTTP*; *NAV2*; *NECAP2*; *OTOGL*; *PPM1D*; *SAFB2*; *SCD*; *SFXN2*; *SLC9A8*; *STARD8*; *TIMP1*; *TRAK2*
IRF2	25	*ADPRH*; *ARHGEF37*; *COX15*; *DFFA*; *EIF1B*; *FGF10*; *FGG*; *GALR1*; *GPR35*; *HABP2*; *HNMT*; *IMPAD1*; *ITGAL*; *LIX1*; *MTTP*; *NEGR1*; *PEX6*; *PPM1D*; *PTX3*; *RGS9*; *RMND5B*; *SCD*; *SGSM1*; *TTPAL*; *ZFPL1*
PPARA	3	*BAZ1B*; *RARRES1*; *TIMP1*
PPARG	26	*ADCK5*; *AGK*; *C16ORF58*; *CNST*; *CORO1A*; *CYB561D1*; *ECE1*; *FUS*; *GPR68*; *HABP2*; *IP6K2*; *ITGAL*; *KANK3*; *KIAA0355*; *MYO3B*; *NECAP2*; *PDE4D*; *PITX2*; *PRMT3*; *PTX3*; *RARRES1*; *RYK*; *SCD*; *TMEM39B*; *VPS25*; *ZRANB1*
RARA	19	*ADCY10*; *AURKB*; *CORO1A*; *GPR68*; *HABP2*; *ITGAL*; *KANK3*; *KIAA0355*; *MTTP*; *MYO3B*; *NCF1*; *NECAP2*; *PRMT3*; *PTX3*; *RMND5B*; *SCD*; *SH2D3A*; *TMEM42*; *TTPAL*
RXRA	14	*ACTC1*; *BAZ1B*; *CORO1A*; *EXOSC1*; *FGF10*; *GPIHBP1*; *GSTO2*; *KIAA0355*; *PEX6*; *PRMT3*; *PYCARD*; *RARRES1*; *TMEM42*; *VPS25*
SREBF1	34	*AADAC*; *ABCA1*; *ACTC1*; *CACNA1l*; *CDK13*; *COL17A1*; *CORO1A*; *DFFA*; *DNER*; *ECE1*; *FGG*; *FUS*; *HK2*; *IP6K2*; *KANK3*; *LIX1*; *LRTM1*; *MORN4*; *MTTP*; *MVK*; *NAV2*; *NECAP2*; *PDE4D*; *PDE6D*; *PDZD8*; *PRMT3*; *RARRES1*; *RASA3*; *RGS9*; *SH2D3A*; *TIMP1*; *TMPRSS13*; *TNK2*; *ZFPL1*
SREBF2	10	*AADAC*; *ARHGEF37*; *IGFBP4*; *IP6K2*; *ITGB2*; *LZIC*; *NAV2*; *SLC12A9*; *TNK2*; *VPS25*

### Quantitative PCR validation of RNA-seq results

Ten relevant genes were selected from the DEG list for the validation experiment based on the FC ratios and the functional annotation of the gene. Efficient qPCR assays were established for 8 out of the 10 selected genes. For all genes (Table 6), the fold-change ratios between groups were consistent in both assays. Expression differences between groups were significant for all genes tested (*P* < 0.05) except for *ABCA1* and *MTTP*. In addition, in most cases the expression ratios were lower in the qPCR experiment than in RNA-seq, which might be due to the higher background noise of the qPCR assay. Altogether, validation of RNA-seq data by qPCR showed a high correspondence between both analyses, confirming differential expression for 8 out of 10 group comparisons (Table 6).

**Table 6:** Validation of differentially expressed genes (DEG) by quantitative PCR

		RNA-seq	qPCR	
DEG between	Gene	FC^**a**^	FC^**a**^	***p***-value
*SCD* genotypes (TT vs CC)	*SCD*	2.15	3.00	0.003
diets in *SCD*_TT pigs (TT_VA+ vs TT_VA-)	*ABCA1*	1.85	1.41	n.s.
	*FGF10*	5.30	1.95	0.03
	*PPARA*	0.63	0.78	0.05
diets in *SCD*_CC pigs (CC_VA+ vs CC_VA-)	*TNMD*	131.44	253.75	0.04
	*OTOR*	33.01	51.30	0.02
	*CILP2*	34.57	14.21	0.03
*SCD* genotypes under the VA+ diet (TT_VA+ vs CC_VA+)	*MTTP*	2.63	1.97	n.s.
	*FGF10*	6.55	2.08	0.03
	*SCD*	2.84	4.05	0.03

^a^ FC, fold-change ratio between groups as indicated in the first column

## Discussion

In the present study, carcasses from pigs fed the VA- diet were 3.8 kg heavier than those fed the VA+ diet. Despite of this, dietary vitamin A had no effect on carcass lean content, which agreed with previous studies in different pig lines fed with restricted or elevated vitamin A [5–11].

Regarding meat quality traits, we observed no differences in IMF between dietary vitamin A treatments in none of the three muscles analysed. The effect of dietary vitamin A on IMF deposition is particularly controversial in pigs. Several authors reported that a dietary reduction of vitamin A impacted the IMF content in *m. gluteus medius* [8], *m. longissimus thoracis* [9, 11] and *m. semimembranosus* [7] in pigs of different genetic lines, although the direction and magnitude of the changes were very variable. For instance, Olivares et al. [11] investigated the effect of supplementing the feed for 11 weeks with 100-fold the vitamin A daily recommendations of the National Research Council. This extra supplementation raised IMF content in Duroc-sired hybrid pigs but not in Large White × Landrace animals. In contrast, removing vitamin A supplements from the formulation also increased IMF content in Iberian [6] and Large White × Landrace [10] pigs. In a previous work with the same Duroc line used in this study, the complete removal of vitamin A from the diet the last 30 days of fattening also resulted in higher IMF content [8].

The experimental conditions, the duration of vitamin A restriction, age and genetic type are several factors that can explain the poor consistency of the results. Retinoic acid, an active metabolite of vitamin A, regulates the adipogenic differentiation of fibroblasts into adipocytes in intramuscular adipose tissue and, for this reason, dietary vitamin A could impact IMF deposition [4]. However, given the opposite effect of retinoic acid in early (positive) and late (negative) differentiation of adipocytes [4], the time and duration of the dietary restriction might be critical to have relevant consequences in the pig.

As opposed to other experiments [5–9, 11], in our study the FA composition was not directly affected by the dietary vitamin A. In general, most studies have detected an increase in the desaturation index (MUFA/SFA) when dietary vitamin A was restricted. We detected this effect as an interaction between the diet and the *SCD* genotype of the pigs. In this sense, vitamin A supplementation promoted FA desaturation and increased C18:1n9 content at the expense of C18:0 in *SCD* CC pigs but not in TT and TC pigs (Fig. 1). This trend was consistent on the three muscles analysed but was more evident in *gluteus medius*, probably due to the higher IMF content of this muscle and the larger number of pigs sampled. The effect of the *SCD* genotype on MUFA content and FA desaturation indexes has been described before [14, 33, 34]. The TT pigs have more oleic acid (C18:1n9), MUFA and, consequently, C16:1/C16:0, C18:1n7/C18:0, C18:1n9/C18:0 and MUFA/SFA than CC pigs, which we observed in both diets, but the differences were more evident in pigs fed the vitamin A-restricted diet. This interaction reinforces the relationship between vitamin A and SCD activity, with the CC pigs being more sensitive to the effect of vitamin A. In a previous work, we described a genetic interaction between dietary vitamin A and the *SCD* genotype for liver levels of all-*trans*-retinol and all-*trans*-5-6-epoxy retinoic acid, the two most abundant forms of bioactive vitamin A [8]. The effect of the diet on the level of these two compounds was only evidenced in CC pigs, paralleling our current results on FA desaturation indexes. Note that the *SCD* rs80912566 polymorphism is not present in all pig breeds. The T variant was fixed in the Iberian, Piétrain and Landrace lines tested in [14] but is segregating in Large White lines [14, 35], which might partly explain the inconsistent results from previous studies.

Despite the small impact that the dietary vitamin A had on the phenotype of the pigs, we observed significant changes at the transcriptome level. The highest number of DEGs were detected when diets by *SCD* or *SCD* by diets groups were compared. Although most DEG were protein-coding genes, about 15% of DEGs represented transcripts from lncRNA genes, which are still not well annotated in pigs, and little is still know about their functional relevance. In fact, the highest differences in gene expression were mainly lncRNA genes. Other groups have described similar situations where dietary treatments have little effect in the phenotype but profound effects at the transcriptome levels (for instance [36] and [37] for carbohydrate and oleic-enriched diets in pigs, or [38] with pigs on different fasting periods).

The most striking result of our data is the high number of DEGs when the interaction between diets and *SCD* genotype was considered. On one hand, the vitamin A supplement changed the expression of 162 genes in TT pigs but only 26 genes in CC animals, with little overlap. On the other hand, the VA+ diet induced 73 DEG between pigs of opposite *SCD* genotypes in contrast with only 8 DEG when the pigs where fed the VA- diet. *SCD*-TT pigs have more MUFA and less SFA in muscle samples, which include adipocytes, myocytes, and fibroblasts. Oleic acid (C18:1n9) is a potent activator of several transcription cascades, including preadipocyte differentiation [39] and mammary gland FA biosynthesis [40, 41]. Targeted disruption of *SCD* has significant consequences in the health of knockout mice, which are driven to general hypermetabolism and stimulation of FA beta-oxidation. Consequently, *SCD* knockout mice are protected from fat diet-induced obesity [42, 43]. In our study, the difference of *SCD* expression was 2.84-fold in pigs fed the VA+ diet but *SCD* expression did not differ between genotypes in pigs fed the VA- diet ([14] and Additional file 1: Table S6.A), which agrees with differences in liver *SCD* expression due to dietary vitamin A levels, reported in a previous experiment [8]. Overall, our animal material allows studying the long-term effect of higher MUFA content in muscle and fat tissues and its interaction with dietary retinoic precursors.

Retinol and retinoic acid, the biologically active compounds of vitamin A, mediate their function normally through specific retinoid receptors, which belong to the ligand-dependent transcription factors superfamily of nuclear receptors, although receptor-independent effects are also known [44]. Upon binding to retinoids, the nuclear receptors RXR, RAR, HNF4A can form heterodimers with ligand-mediated co-regulators PPAR and HNF1A or members of the NR2 nuclear repressors. Most of these transcription factors were identified as main regulators of 104 of the DEGs detected in this experiment. Retinol-mediated gene repression and transactivation have wide effects on cell proliferation, differentiation, cell adhesion, and apoptosis in different cell types, immunity, male and female reproduction, embryonic development, and barrier integrity [45]. This was also reflected in our data (Additional file 1: Tables S7 and S8), as many of these processes were included in the functional analysis of the DEGs.

Among the DEGs, it is interesting to highlight *ABCA1*, a cholesterol transporter involved in maintaining cholesterol homeostasis and lipid metabolism [46]. In pigs, *ABCA1* overexpression is directly associated with an increase in HDL levels [46, 47] and polymorphisms on this gene have been associated with atherosclerosis risk score [48]. *ABCA1* has been used as an epigenetic marker for evaluating meat quality in chicken [49] and it has been associated with beef tenderness and FA composition [50] but information in relation to pork quality is still lacking. A gene with a similar function is *MVK*, a mevalonate kinase that catalyses an early step in cholesterol biosynthesis and is associated with HDL cholesterol [51]. This SREBP2-responsive gene has been appointed as a candidate gene for fat deposition in broilers [52, 53]. Unfortunately, we were not able to validate the differential expression of these two genes by qPCR analysis. On the other hand, a group of four genes encoding extracellular matrix proteins (*COMP*, *CILP2*, *OTOR* and *TNMD*) were very highly expressed (30 to 130-fold) in CC pigs fed the VA+ diet with respect to the VA- diet. Three of these genes have been individually investigated in relation with adipocyte function (*CILP2* [54]) and differentiation (*TNMD* [55, 56]; *COMP* [57]) but not together. Several other genes are also well known in relation to adipose tissue differentiation, such as *FGF10* and *MTTP.* In chicken, muscle fibroblast growth factor 10 (*FGF10*) expression correlates with IMF content [58].. We were able to validate the higher expression of *FGF10* in TT pigs than in CC pigs fed the VA+ diet (6.55-fold by RNA-seq analysis and 2.08-fold by qPCR assay) and in the TT pigs fed the VA+ diet vs the VA- diet (5.30 vs 1.95-fold by RNA-seq and qPCR analysis, respectively). Microsomal triglyceride transfer protein (*MTTP*) is a protein essential to transport triglycerides from the endoplasmic reticulum membrane to lipid droplets [59]. Polymorphisms in this gene have been associated with changes in FA composition in pork [60, 61] but there is so far no report on the effect on the expression of the gene on pork quality.

Another interesting DEG is *PPARA*, which, as mentioned before, encodes a member of the retinol receptor family whose expression is inhibited by dietary vitamin A intake [62]. In agreement with this, in our study *PPARA* expression increased by 1.6-fold in TT pigs fed the VA- diet. The PPARα transcription factor promotes de novo lipogenesis, FA storage and improves glycogen synthesis [63]. In pigs, *PPARA* can be used as a genetic marker for overall adipose tissue accumulation, as polymorphisms in the 3′ untranslated region (UTR) are associated with changes in backfat and IMF deposition [64]. The increase in *PPARA* expression agrees with the general observation that restricted dietary vitamin A promotes adipogenesis and fat deposition, although we were not able to capture this effect in the phenotype of our pigs.

## Conclusions

In our experiment, restricting the dietary vitamin A supplement during the fattening period did not contribute to improving IMF content despite relevant changes in muscle gene expression, both in coding and non-coding genes. However, there was a clear interaction between the *SCD* genotype and the dietary vitamin A for FA composition, which affected the desaturation rate of IMF, particularly in *SCD* CC pigs in opposite directions to *SCD* TT and TC pigs. Taken together, our results indicate that a restriction of dietary vitamin A might be a strategy to enhance monounsaturated FA content only in pigs carrying the *SCD*-T allele.

## Supplementary Information

**Additional file 1.**

**Additional file 2.**

## Data Availability

Data that support the findings of this study are available within the article and Supplementary Information, or from the authors upon reasonable request. Sequencing files are available from NCBI-GEO with access number GSE183909.

## Abbreviations

DEG: Differentially expressed genes
IMF: Intramuscular fat
MUFA: Monounsaturated fatty acids
PUFA: Polyunsaturated fatty acids
RXR: Retinoid receptors
RAR: Retinoic acid receptor
SCD: Stearoyl-CoA desaturase
SE: Standard error
SFA: Saturated fatty acids
SNP: Single nucleotide polymorphism
