# Supplementary material for: Transcriptome shifts triggered by vitamin A and SCD genotype interaction in Duroc pigs
Source: BMC Genomics. 2022 Jan 7;23:16. doi: 10.1186/s12864-021-08244-3 (PMC8739656; doi:10.1186/s12864-021-08244-3)
Supplement: Supplementary file 2 — Additional file 2. [file 12864_2021_8244_MOESM2_ESM.pdf]

Transcriptome shifts triggered by vitamin A and *SCD* genotype interaction in Duroc pigs

Emma Solé[1], Rayner González-Prendes[1,2], Yelyzaveta Oliinychenko[3], Marc Tor[1], Roger Ros-Freixedes[1], Joan Estany[1] and Ramona N. Pena[1*]

Figure S1. Effect of *SCD* rs80912566 genotype and diet on four fatty acid saturation indexes. A. *m. longissimus thoracis*. **B.** *m. semimembranosus*. Diets with (VA+) and without (VA-) vitamin A supplement in the feed formulation. Within each panel, bars not connected with the same letter differ at P<0.05. Errors bars are SE.

Figure S2. Gene network analysis of differentially expressed genes as determined by RNA Seq, The full list of 202 unique HGNC gene name identifiers was analysed using the STRING App of Cytoscape.

Figure S3. Close up look at the two main gene networks detected by the STRING App of Cytoscape. **A.** membrane-bound vesicle-related genes; **B.** Genes involved in fatty acid beta-oxidation in peroxisomes.

Figure S1.

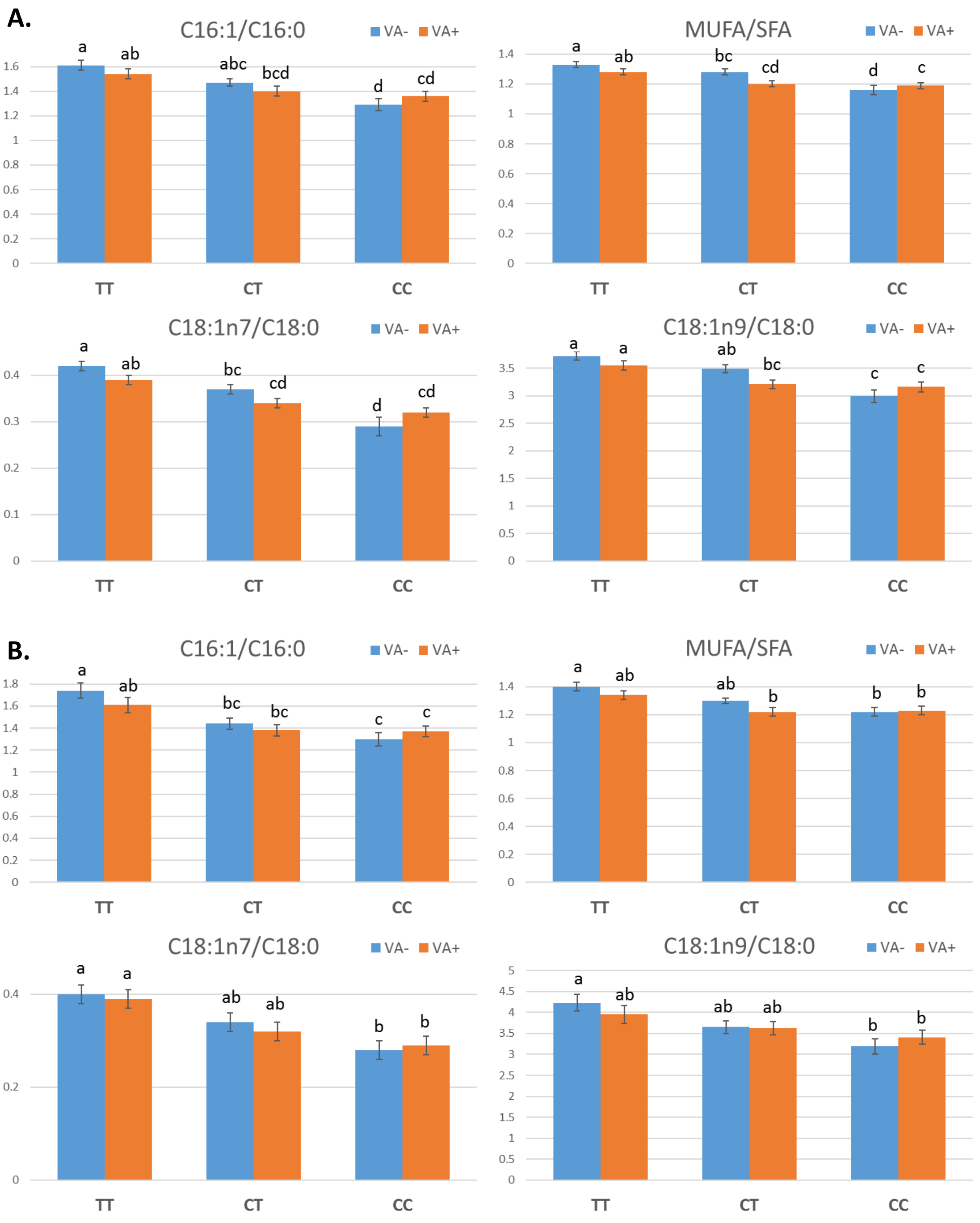
A.
C16:1/C16:0
VA- VA+
a ab abc bcd d cd
TT CT CC
MUFA/SFA
VA- VA+
a ab bc cd d c
TT CT CC
C18:1n7/C18:0
VA- VA+
a ab bc cd d cd
TT CT CC
C18:1n9/C18:0
VA- VA+
a a ab bc c c
TT CT CC
B.
C16:1/C16:0
VA- VA+
a ab bc bc c c
TT CT CC
MUFA/SFA
VA- VA+
a ab ab b b b
TT CT CC
C18:1n7/C18:0
VA- VA+
a a ab ab b b
TT CT CC
C18:1n9/C18:0
VA- VA+
a ab ab ab b b
TT CT CC

Figure S2.

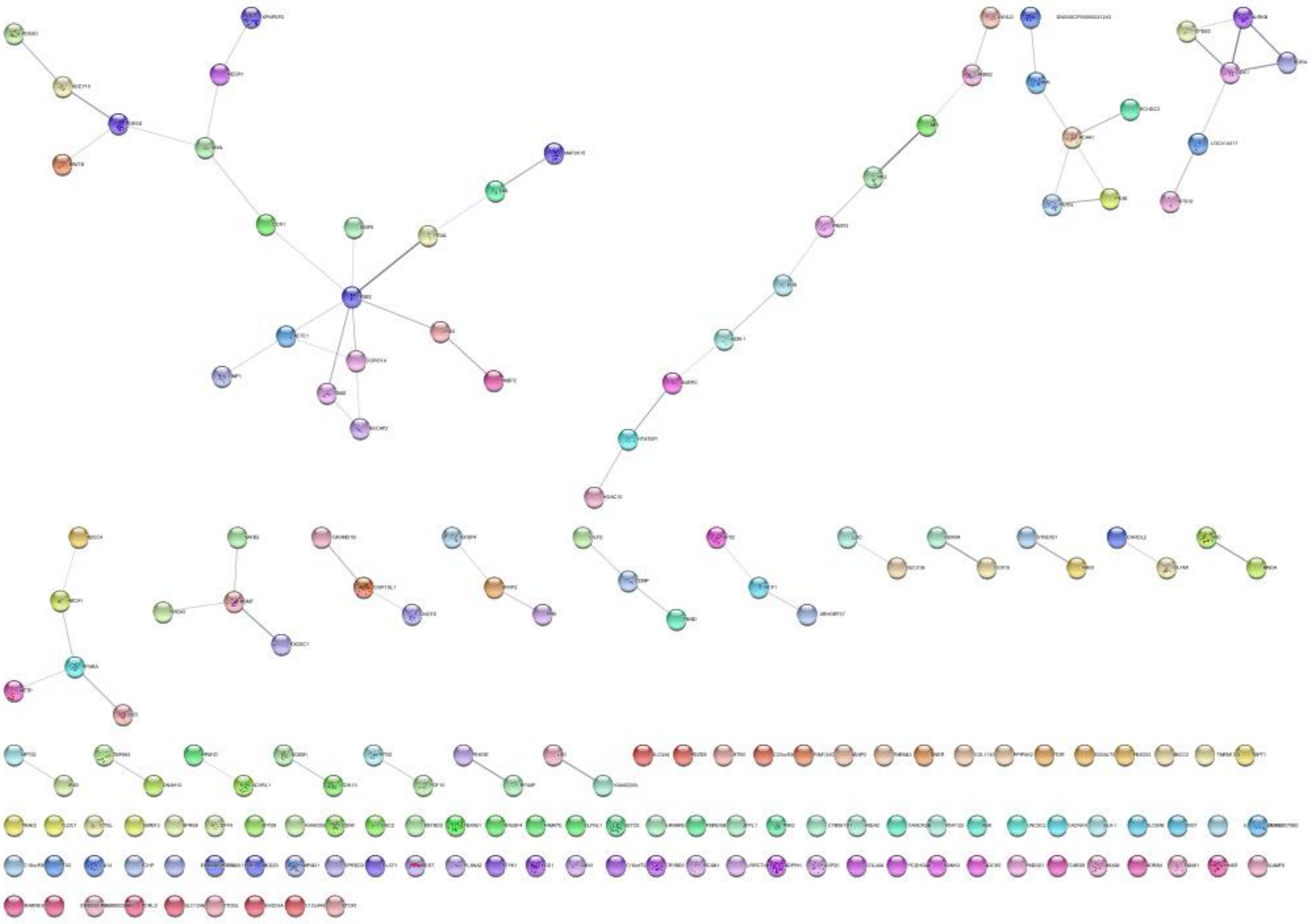

Figure S3.

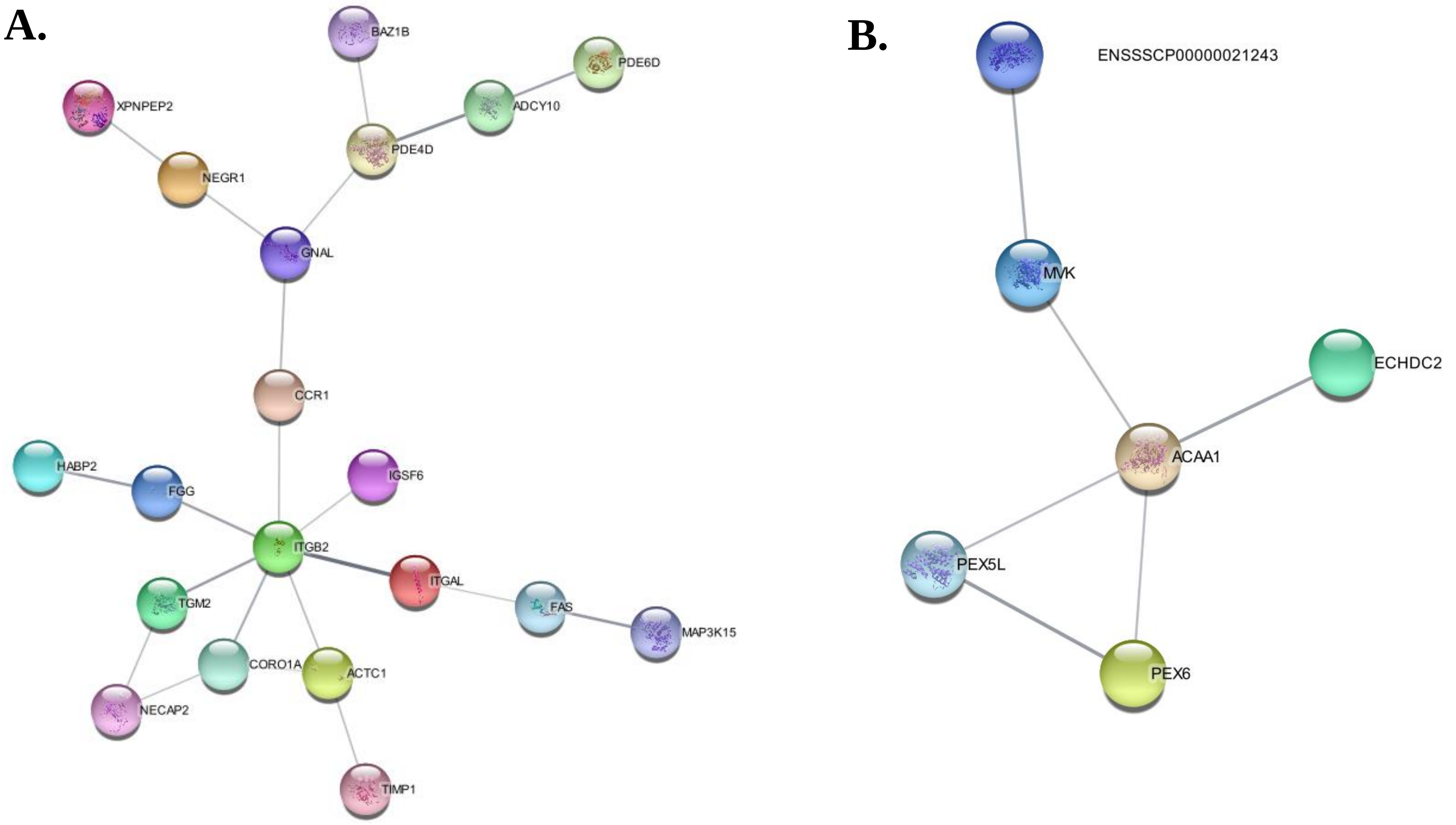
A.
BAZ1B
PDE6D
XPNPEP2
ADCY10
PDE4D
NEGR1
GNAL
CCR1
HABP2
FGG
IGSF6
ITGB2
ITGAL
FAS
MAP3K15
TGM2
CORO1A
ACTC1
NECAP2
TIMP1
B.
ENSSSCP00000021243
MVK
ECHDC2
ACAA1
PEX5L
PEX6
